# Metabolomic and Sensory Insights into the Aging Mechanism of Ripened Pu-Erh Tea over Nine Years

**DOI:** 10.3390/molecules31111937

**Published:** 2026-06-03

**Authors:** Nianguo Bo, Teng Wang, Qiuyue Chen, Yiqing Guan, Dihan Yang, Fan Yang, Hongyan Gao, Xiaying Tao, Ping Liang, Bei Cai, Guanghong Pan, Yingling Zhou, Ming Zhao

**Affiliations:** 1 The Key Laboratory of Agricultural Microbiome of Yunnan Province, The National & Local Joint Engineering Research Center on Germplasm Innovation & Utilization of Chinese Medicinal Materials in Southwestern China, College of Agronomy and Biotechnology, Yunnan Agricultural University, Kunming 650201, China; bobo0800@yeah.net (N.B.); tengwww@126.com (T.W.); 13669799142@163.com (Q.C.); dxcxygyq@126.com (Y.G.); 15680783708@163.com (D.Y.); 18887400747@163.com (F.Y.); 14787855147@163.com (H.G.); xiayingtao666666@163.com (X.T.); 13337259484@163.com (P.L.); 18708605279@163.com (B.C.); 15752328598@163.com (G.P.); 15828552660@163.com (Y.Z.); 2 College of Tea Science, Yunnan Agricultural University, Kunming 650201, China

**Keywords:** ripened pu-erh tea, aging, storage, volatile organic compounds, sensory evaluation, K-means clustering, relative odor activity value (ROAV), methoxybenzenes, chemical markers

## Abstract

Storage duration critically shapes the characteristic sweet and mellow quality of ripened pu-erh tea (RPT), yet the underlying chemical mechanisms remain poorly understood. This study investigated the sensory and chemical evolution of a representative commercial RPT product across a nine-year storage gradient (1, 3, 5, 7, and 9 years) by integrating Quantitative Descriptive Analysis (QDA), chromaticity measurement, targeted quantification of 42 non-volatile components, and Headspace Gas Chromatography–Mass Spectrometry (HS-GC-MS) volatilomics with multivariate statistical modeling. Prolonged storage drove systematic sensory maturation: the stale aroma gradually purified, and the taste profile transitioned significantly from heavy and mellow to sweet and mellow (*p* < 0.05), accompanied by a deepening infusion color with increased redness and yellowness indices. Targeted chemical profiling revealed significant decreases in total polyphenols and astringent esterified catechins, particularly epicatechin gallate (ECG) and epigallocatechin gallate (EGCG) (*p* < 0.05), while theabrownins remained stable and soluble sugars peaked at intermediate storage stages. Pearson correlation analysis linked these chemical shifts to sensory perception, with enhanced sweetness, mouthfeel thickness strongly associated with reduced monomeric catechins and free amino acids (*p* < 0.001). Volatilomics combined with K-means clustering and relative odor activity value (ROAV) analysis revealed a dual mechanism of flavor refinement: progressive accumulation and increasing odor activity of aged aroma markers (1,2,3-trimethoxybenzene, *β*-ionone) coupled with systematic attenuation of pungent acids and grassy aldehydes. These findings, based on a single, standardized commercial product, elucidate the chemical-sensory foundation of the sweet and mellow profile in aged RPT and provide candidate markers and a transferable analytical framework for quality assessment of stored teas.

## 1. Introduction

Pu-erh tea, a prestigious Chinese Geographical Indication product and Intangible Cultural Heritage derived from *Camellia sinensis* var. *assamica* in Yunnan, China [1], is categorized into raw (RwPT) and ripened (RPT) types based on their distinct processing technologies [2,3]. Unlike RwPT, which is processed directly from sun-dried leaves, RPT is a characteristic dark tea that relies on microbial pile-fermentation as the critical stage for its unique quality formation [4]. RPT is valued for its sensory properties and health potential, which primarily functions as a metabolic regulator, delivering hypolipidemic, hypoglycemic, and anti-inflammatory benefits by profoundly remodeling the gut microbiota to enhance intestinal barrier function. This microbial modulation activates key signaling pathways, including the SCFA-mediated PPARγ/AMPK and gut-liver FXR-FGF15 axes, which systemically improve lipid and glucose homeostasis while restoring redox-inflammatory balance [5]. While pile-fermentation establishes the initial chemical profile, the subsequent long-term storage is widely regarded as a post-fermentation stage, where residual microbial activity and thermostable enzymes continue to drive slow but profound metabolic shifts. Recent studies have demonstrated that specific microbial consortia, particularly *Aspergillus* and *Bacillus* species, remain metabolically active throughout storage and play key roles in the oxidative polymerization of catechins and the biosynthesis of methoxybenzenes that characterize aged RPT aroma [6,7].

Distinct from green or black teas, which are typically preferred for their freshness, pu-erh tea relies on storage as a fundamental post-production stage for pu-erh tea. During this period, complex transformations of metabolites occur, enhancing sensory quality and commercial value. Previous studies on RwPT have extensively documented these shifts. For instance, Liu et al. [8] and Guo et al. [9] observed a transition from floral-fruity notes to smoky and stale aromas over time. Chen et al. [10]. identified key methoxybenzene derivatives as aging markers using HS-SPME/GC × GC-QTOFMS, while others established volatile fingerprints via multi-platform strategies [11,12]. Notably, Xu et al. [13] reported that the third and eighth years of storage represent critical quality inflection points for RwPT. However, these milestones were identified using relatively low-frequency sampling, leaving the transitional dynamics between these key years—and whether similar inflection points exist for RPT largely unexplored.

Recent progress in RPT storage research has begun to unravel the complex dynamic shifts in its metabolite profiles. Li et al. [6] utilized integrated volatolomics and metabolomics to demonstrate that catechins and caffeine decrease during the RPT storage process, while the volatile profile evolves through a decrease in aldehydes and alcohols alongside an increase in hydrocarbons, contributing to the characteristic stale aroma. Moreover, Wang et al. [14] elucidated the aging mechanism of RPT aroma by identifying 114 volatile compounds and their potential metabolic pathways. Their findings revealed that methylphenols derived from precursors such as butylphenols and butylated hydroxytoluene act as indispensable intermediates in the synthesis of methoxybenzenes via caffeine metabolism and hydrocarbon degradation pathways. Yang et al. [15] found that, comparing RPT from Lancang and Shuangjiang (Lincang) stored for 3 years with those stored for 8 years, the content of methoxybenzenes in the 8-year samples increased twofold, while alcohols gradually decreased. Notably, Xie et al. [16] noted a decline in specific terpenoids alongside a significant rise in phytone. Crucially, these chemical transformations are not merely spontaneous oxidations but are significantly mediated by the persistent metabolic activity of microorganisms. Li et al. [6] demonstrated that *Aspergillus* and *Bacillus* remain the dominant genera throughout 0–15 years of RPT storage, playing key roles in metabolite decomposition and synthesis that directly influence the flavor and aroma characteristics of aged RPT. Recent metagenomic evidence further indicates that these microbial consortia facilitate the O-methylation of phenolic precursors into the methoxybenzene derivatives that define aged RPT aroma [7]. Despite these insights, Zhao et al. [17] examined RPT five-year storage gradient and observed a general decline in water extracts, total polyphenols, and theabrownins (TBs), which correlated with reduced antioxidant activities.

Despite these valuable insights, current research on RPT storage presents three notable limitations. First, conclusions regarding key chemical trajectories remain inconsistent, particularly concerning the further accumulation or stabilization of theabrownins (TBs) [13,18]. Second, most studies investigate sensory attributes or chemical profiles in isolation. Importantly, the literature has largely neglected the application of the Odor Activity Value (OAV) or Relative OAV (ROAV) concept—a well-established approach for quantitatively linking volatile concentrations to actual sensory impact by accounting for individual odor thresholds [19]. In recent pu-erh tea research, ROAV-based approaches have been successfully applied to identify key odor-active compounds. Chen et al. [20]. identified 19 odor-active compounds through ROAV and Pearson correlation analysis, and aroma recombination experiments successfully reproduced the characteristic jujube-like aroma of pu-erh tea. Similarly, Yang et al. [15] applied OAV analysis to RPT from different producing areas and storage durations, demonstrating that 1,2,3-trimethoxybenzene exhibited OAV > 100 and was the key aroma component of aged RPT. Without this quantitative bridge, the functional contribution of specific dynamic VOCs to the perceived stale aroma remains speculative. Third, few studies have applied an integrated analytical framework to a single standardized commercial product across a high-resolution, multi-year gradient.

To address these gaps, this study integrated modified QDA, CIELAB colorimetry, and HPLC-based quantification of 42 non-volatile metabolites with HS-GC-MS-based volatilomics, underpinned by multivariate statistical modeling. We aimed to systematically elucidate the co-evolution of sensory attributes and chemical profiles in a representative commercial RPT (Laotongzhi 9978) over a nine-year storage period. Specifically, the research sought to delineate temporal sensory trajectories, identify the primary chemical markers driving flavor maturation, and establish a robust quantitative framework linking chemical transformations to the evolving quality of aged RPT.

## 2. Results and Discussion

### 2.1. Sensory Evaluation Result and Change in Tea Infusion Color

**Figure 1:**
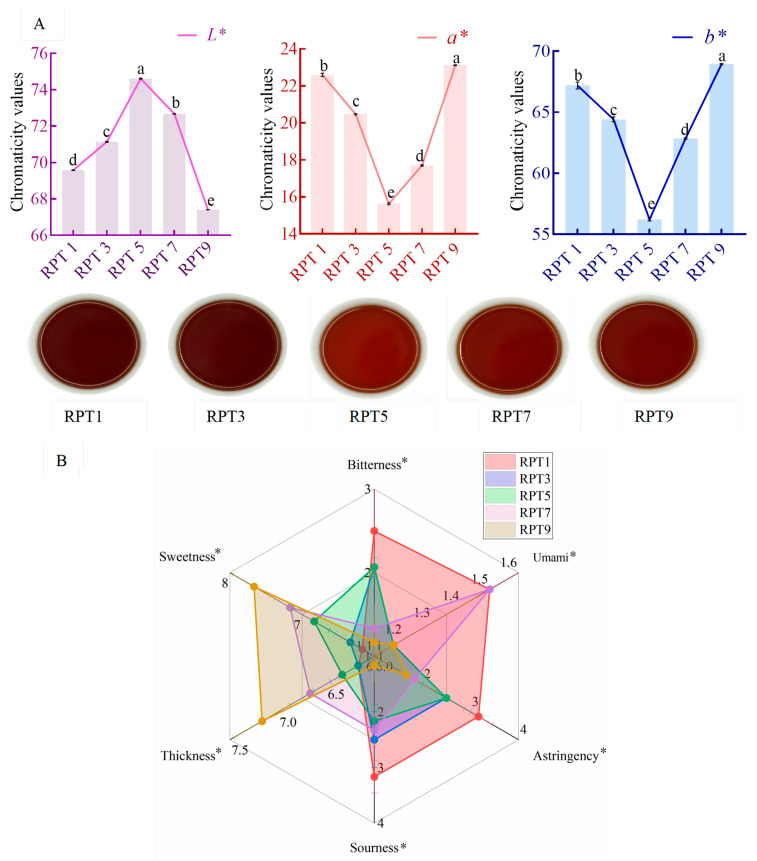
Changes in infusion color and sensory attribute intensities of RPT during storage. (**A**) Changes in L*, a*, b* values of tea infusions across different storage years. Error bars represent standard deviations (*n* = 3). Different lowercase letters above bars indicate statistically significant differences at *p* < 0.05 based on one-way ANOVA followed by Duncan’s multiple range test. (**B**) Radar plots of QDA sensory scores. Attributes include taste (bitterness, sweetness, sourness, umami) and mouthfeel (astringency, thickness). * *p* < 0.05.

To elucidate the quality dynamics of RPT during storage, sensory attributes and chromaticity parameters were analyzed across five vintage gradients (RPT1–RPT9) (Table 1, Figure 1). As shown in Table 1, the RPT samples across nine years maintained a consistent appearance profile characterized as reddish brown, slightly tippy, and with more bloom. The aroma profile evolved from a stale aroma and woody aroma that approached pure in the early stages (RPT1–RPT3) to a stale aroma and woody aroma that were pure and normal in samples stored for five years or longer. These changes in aromatic characteristics are consistent with the findings reported by Xie et al. [16]. Similar stage-specific transitions have been observed in raw pu-erh tea, where the third and eighth years of storage were identified as critical turning points for flavor evolution [21].

Concurrently, the taste profile transitioned from heavy and mellow (RPT1) and mellow and thick (RPT3) to a refined sweet and mellow sensation by RPT9, indicating that prolonged storage enhances the characteristic taste profile’s sweetness and mouth feel thickness of the tea. To further quantify these sensory shifts, QDA approach was employed (Figure 1B, Table S1). The radar plot reveals that as storage time progressed, the scores for sweetness (6.17~7.67) and thickness (6.17~7.17) significantly increased (*p* < 0.05), while those for astringency (3.17~1.67) and bitterness (2.5~1.17) significantly decreased (*p* < 0.05). This shift quantifies the transition toward a more mellow and balanced profile.

Visual inspection of the infusion revealed a color shift from reddish brown and bright (RPT1–RPT3) to reddish and bright (RPT5), eventually deepening to heavy reddish and bright (RPT7–RPT9). This trend aligns with findings by Zhang et al. [22] regarding the color of RPT infusion follows a maturation trend from brownish red and bright to reddish brown and bright, and finally to heavy reddish and bright during the storage process. This visual maturation was quantitatively corroborated by the chromaticity values (L*, a*, b*). The lightness (L*) values (67.4~74.6) indicated high clarity across all stages, peaking at RPT5. Notably, the redness (a*) and yellowness (b*) indices declined during RPT1-5 but increased in RPT5-9. RPT9 reached the maximal values for a* (23.12 ± 0.02) and b* (68.95 ± 0.06). This upward trend in a* and b* values during later storage explicitly links to the development of higher color saturation (chroma) and a more profound reddish-brown hue, confirming that prolonged aging leads to a more visually saturated profile (Figure 1A, Table S2). In summary, storage years increase, RPT quality evolves through the purification of stale aroma, a fluctuating enhancement of liquor saturation, and a taste transition from heavy and mellow to sweet and mellow sensations.

### 2.2. Dynamic Evolution of Characteristic Chemical Components

The sensory evolution of RPT is underpinned by substantial shifts in its chemical profile during storage. To elucidate the molecular basis of the sweet and mellow quality, 42 key components were quantified across the storage gradient (Table 2).

#### 2.2.1. Pigment and Polyphenols Transformation

**Figure 2:**
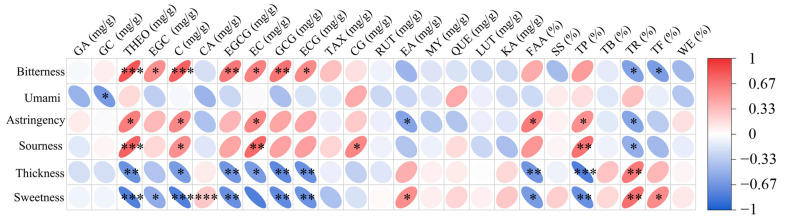
Correlation analysis between QDA scores and characteristic chemical components during RPT storage. Note: The squares represent the direction of the correlation (red: positive; blue: negative). In the elliptical representation, a narrower ellipse and a deeper color indicate a larger absolute correlation coefficient. Statistical significance is denoted as * *p* < 0.05, ** *p* < 0.01, and *** *p* < 0.001.

The chromatic transition of the tea infusion was mirrored by significant fluctuations in color-active constituents. Theabrownins (TB) remained the dominant pigment throughout the aging process, maintaining high levels and peaking in the oldest samples (RPT9, 14.24 ± 0.33%), confirming their role in establishing the characteristic reddish-brown hue. In contrast, thearubigins (TR) showed an increasing trend; they remained low (1.38~2.17%) in early storage but increased significantly by approximately 4.5 fold (*p* < 0.05, from 1.38 ± 0.10% to 6.22 ± 0.48%), coinciding with the deepening of the infusion color. Theabrownins and thearubigins are the primary water-soluble pigments and the main color components in tea infusions. They give the infusions a reddish-brown infusion color [23]. Total polyphenols (TPs) peaking at RPT3 (9.54 ± 0.01%) before significant degradation (*p* < 0.05, Table 1) to reach a minimum in RPT9 (4.41 ± 0.78%). This inverse correlation between declining polyphenols and accumulating polymeric pigments (TR and TB) aligns with the oxidative polymerization mechanism described in aged teas [24]. As illustrated in Figure 2, TP content shows a positive correlation with bitterness scores in the QDA, while exhibiting a significant negative correlation with sweetness scores (*p* < 0.001). Consequently, the substantial reduction in TP content is a primary driver for the increased sweetness observed in RPT samples following storage.

#### 2.2.2. Taste Active Components

The transition toward a sweet and mellow taste profile is chemically substantiated by the dynamics of soluble sugars and amino acids. Soluble sugars (SS) and water extracts (WE), which govern sweetness and viscosity [25], both maximized at RPT5 (8.37 ± 0.61% and 47.54 ± 14.74%, respectively). Correlation analysis further supports this, showing that SS content is positively correlated with the Sweetness scores in the QDA, while exhibiting a non-significant negative correlation with Bitterness scores (Figure 2). This accumulation is likely driven by the hydrolysis of polysaccharides into mono- and oligosaccharides.

Subsequently, free amino acids (FAA), contributors to freshness and umami, significantly declined (*p* < 0.05) from a peak at RPT5 (0.69 ± 0.04%) to a stable low in later stages (0.40 ± 0.02%). Correlation analysis revealed that FAA levels were positively correlated with Bitterness scores while exhibiting a significant negative correlation with Sweetness scores (*p* < 0.05). This reduction in FAA is primarily attributed to their participation in Maillard reactions and oxidative coupling with polyphenols, which are key pathways for flavor stabilization during aging [24]. In early-stage RPT, the high concentration of certain fermentation-related nitrogenous compounds can result in an uncoordinated ‘raw-umami’ or ‘heavy’ sensation that masks the underlying sweetness. As FAA levels stabilize, the increased soluble sugar/amino acid (SS/FAA) ratio reduces this sensory interference, thereby facilitating the transition from a heavy-mellow profile to a more balanced and cleaner taste.

Beyond the sweetness enhancement driven by soluble sugars, recent metabolomic evidence from ultra-long-term Pu-erh tea storage studies suggests that lipid remodeling—the hydrolysis of membrane lipids and triacylglycerols into free fatty acids and monoacylglycerols—may significantly contribute to the characteristic thick and oily mouthfeel of aged RPT [7]. This lipid-derived textural enhancement may operate synergistically with the SS/FAA ratio shift to produce the full sweet and mellow profile observed in our sensory evaluation. Furthermore, the progressive transformation of flavonoid and phenolic acid profiles across the 9-year storage period raises the possibility that Kokumi-tasting γ-glutamyl peptides or similar mouthfulness-enhancing compounds, recently reported in long-term aged teas [18], may have been generated through proteolysis and subsequent enzymatic modification. This chemical refinement allows the aged sweetness to become more prominent, consistent with the sensory maturation pattern where the dissipation of intense primary metabolites leads to a more refined and harmonious palate.

#### 2.2.3. Monomeric Phenolics and Alkaloids

Targeted HPLC profiling revealed distinct degradation patterns for bitterness-related compounds. A clear degradation trend was observed for major monomeric catechins, including epicatechin (EC), epicatechin gallate (ECG), and epigallocatechin gallate (EGCG), all of which peaked at RPT3 before declining significantly (*p* < 0.05). Correlation analysis (Figure 2) further substantiated these findings, showing that Bitterness and Astringency scores were positively correlated with these monomeric catechins, particularly EGCG and ECG (*p* < 0.05 or *p* < 0.01), while Sweetness scores exhibited a highly significant negative correlation with most catechins and THEO (*p* < 0.001). Specifically, EGCG, a primary contributor to bitterness and astringency [26], decreased from 0.82 ± 0.02 mg/g at RPT3 to 0.27 ± 0.03 mg/g at RPT9. This significant reduction in esterified catechins (EGCG and ECG) provides a chemical basis for the observed sensory improvements, specifically the significant reduction (*p* < 0.05) in bitterness and astringency scores during the later stages of storage (Figure 1B). The transition from a harsh or bitter early-stage profile to a more ‘smooth’ and ‘mellow’ aged profile is directly linked to the oxidative polymerization of these monomeric catechins into complex tea pigments, such as theabrownins. This finding is consistent with the results reported by Li et al. [6]. Conversely, caffeine (CA) exhibited high stability throughout the storage period (33.72~37.31 mg/g).

### 2.3. Volatile Compound Metabolic Profile

Volatile organic compounds (VOCs) are the critical determinants of tea aroma [27] and are intrinsically linked to the development of the characteristic stale aroma and the superior storage stability of RPT [28]. To elucidate the chemical basis of aroma evolution during aging, the volatile profiles of RPT samples stored for 1 to 9 years (RPT1–RPT9) were comprehensively characterized via HS-GC-MS, identifying a total of 111 VOCs.

**Figure 3:**
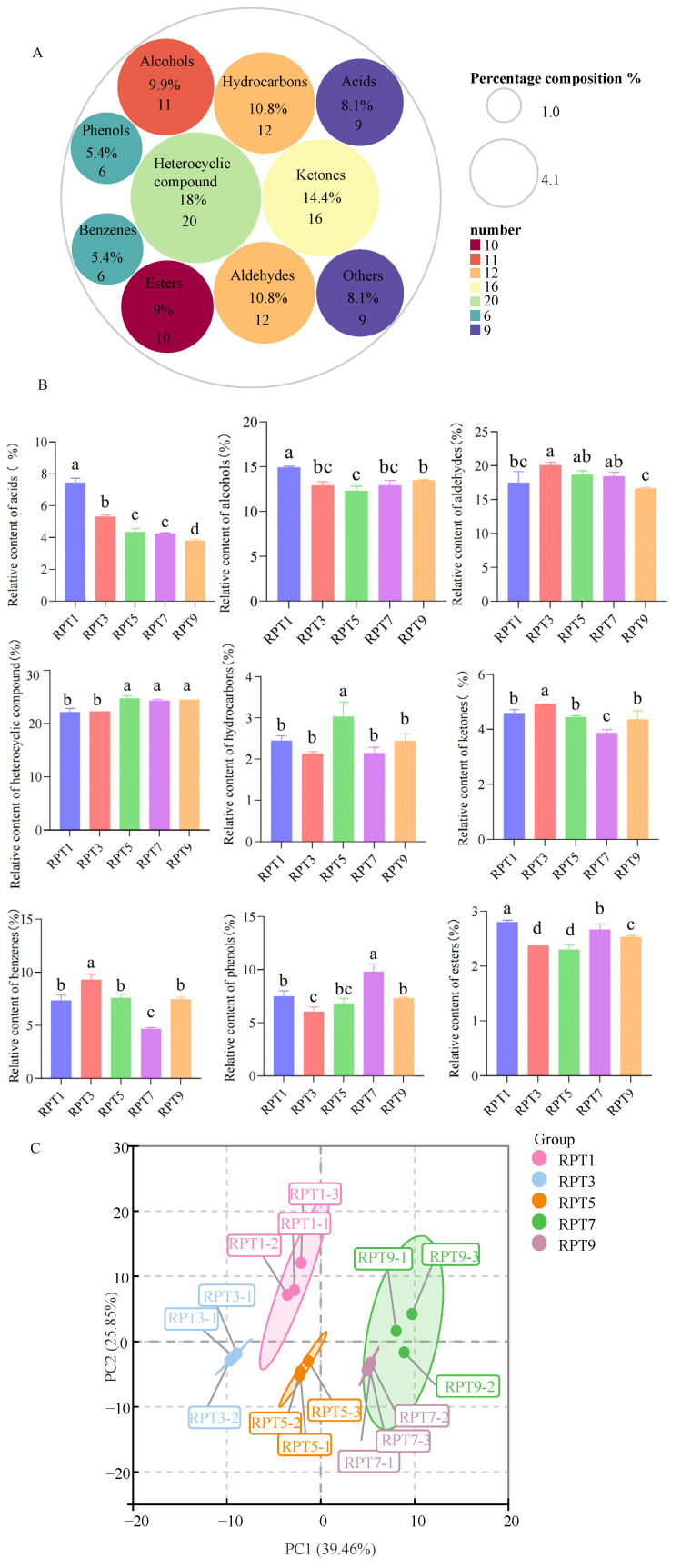
Composition and dynamic changes of volatile compounds. (**A**) Category distribution (percentage and number of compounds) of volatile compounds; (**B**) Relative content changes of volatile compound categories during storage (RPT1 to RPT9); (**C**) PCA analysis of volatile compounds. Different lowercase superscript letters (a, b, c...) within the same column indicate significant differences at *p* < 0.05 based on one-way ANOVA followed by Duncan’s multiple range test.

As detailed in Figure 3A, the volatile profile was dominated by three major chemical classes, which collectively accounted for over 55% of the total volatile content: heterocyclic compounds (20 compounds, 18%), ketones (16 compounds, 14.4%), hydrocarbons (12 compounds, 10.8%), and aldehydes (12 compounds, 10.8%). These classes form the structural foundation of the RPT aroma. The results were consistent with the classes of volatile compounds in RPT reported by Wang et al. [29]. Both their study and this study suggest that hydrocarbons and aldehydes are the most abundant classes of volatile compounds in RPT. Intermediate fractions included alcohols (9.9%), acids (8.1%), and esters (9%), phenols (5.4%), and benzenoids (5.4%), while acids and esters were present at lower abundances (3.14~3.35%).

The temporal dynamics of these volatile classes are illustrated in Figure 3B. Notably, the relative content of acids exhibited a consistent and significant decline from RPT1 to RPT9 (*p* < 0.05). Since short-chain acids are often associated with sour or pungent sensory notes, their steady degradation serves as a chemical prerequisite for the purification of the aromatic profile. This process facilitates the unmasking of the woody and stale aroma attributes characteristic of aged RPT, allowing these stale aroma attributes to become more refined and prominent over time [14]. Conversely, hydrocarbons and heterocyclic compounds displayed non-linear, fluctuating trends, suggesting a dynamic equilibrium between formation and degradation pathways that sustains aroma complexity throughout the aging process.

To evaluate the structural divergence of volatile profiles across the storage gradient, Principal Component Analysis (PCA) was conducted (Figure 3C). The first two principal components, PC1 and PC2, explained 39.5% and 15.5% of the total variance, respectively (cumulative 55.0%). The PCA score plot revealed a clear chronological trajectory: RPT1 was independently distributed in the fourth quadrant; RPT3 and RPT5 clustered near the central region; and long-term stored samples (RPT7 and RPT9) were tightly grouped in the second quadrant. These results demonstrate that storage duration is the decisive factor shaping the volatile landscape of RPT, driving clear stage-specific transitions in its aromatic structure.

#### OPLS-DA Differentiation of Volatile Profiles During Storage

**Figure 4:**
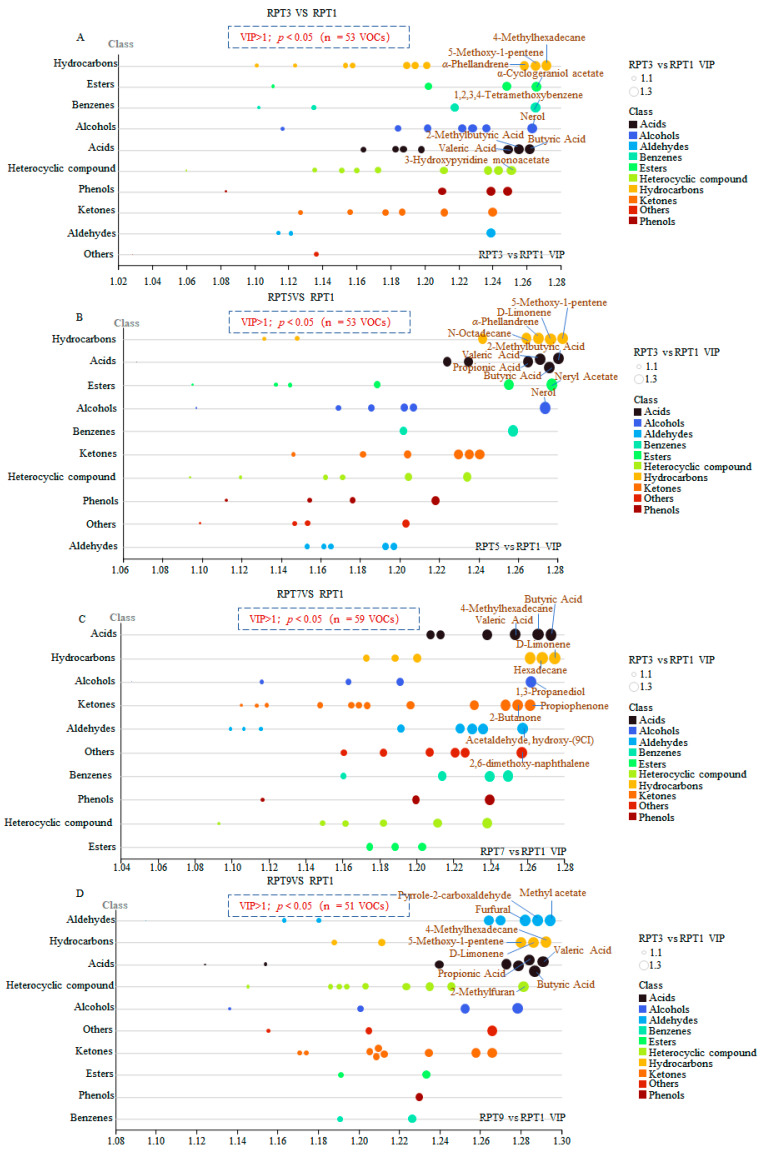
Differential volatile metabolites between RPT3, 5, 7, 9 vs. RPT1. (**A**) RPT3 vs. RPT1 (*n* = 77 VOCs); (**B**) RPT5 vs. RPT1 (*n* = 67 VOCs); (**C**) RPT7 vs. RPT1 (*n* = 79 VOCs); (**D**) RPT9 vs. RPT1 (*n* = 74 VOCs).

Variable Importance in Projection (VIP) analysis was performed to pinpoint the core chemical markers driving the flavor evolution of Ripened pu-erh tea (RPT) across storage durations. Based on the rigorous criteria of VIP > 1 and *p* < 0.05, a total of 53, 53, 59, and 51 differential volatile organic compounds (VOCs) were identified in RPT3, RPT5, RPT7, and RPT9 relative to RPT1, respectively (Figure 4).

These discriminants were predominantly distributed across acids, hydrocarbons, alcohols, aldehydes, and heterocyclic compounds, reflecting a complex multi-class chemical shift during the post-fermentation aging process. Short-chain fatty acids (SCFAs), specifically butyric acid and valeric acid, consistently emerged as top-ranking discriminants across all four aging stages, with VIP scores exhibiting a progressive upward trend that peaked in RPT9 at 1.287 and 1.290, respectively. The sustained high VIP contribution of these organic acids underscores their pivotal role as longitudinal markers, bridging the transition from the aggressive pungency of new ripened tea to the more structured, mellow acidic backbone characteristic of aged samples.

Notably, the early-to-mid stages (RPT3 and RPT5) were characterized by a flavor reconstruction phase, where the persistence of 1,2,3,4-tetramethoxybenzene (VIP = 1.265) in RPT3 reflected residual pile-fermentation characteristics [30]. As the storage duration extended to the mid-to-late stages, the chemical profile underwent a profound shift toward more stable oxidative products and Maillard-derived compounds. In RPT7, the emergence of ketones such as propiophenone and 2-butanone (VIP > 1.25), along with the sustained high contribution of D-limonene (VIP = 1.270), indicated an intensification of oxidative flux that modulates the tea’s aromatic depth. This transition culminated in RPT9, which exhibited the most distinct aged chemical signature. Methyl acetate (VIP = 1.292) and 4-methylhexadecane (VIP = 1.290) ranked as the most influential markers in this final stage, followed by critical stale aroma drivers including pyrrole-2-carboxaldehyde (VIP = 1.288) and furfural (VIP = 1.285). These nitrogen- and oxygen-containing heterocyclic compounds, typically derived from the slow degradation of polysaccharides and amino acid condensation over years of storage, typically impart caramel and roasted nuances [19], providing a robust chemical basis for the enhanced wood-like, caramel, and stale sensory attributes observed in long-term aged RPT. Furthermore, the interplay between diminishing terpene-derived floral notes, such as nerol and *α*-phellandrene, and the rising concentration of high-VIP aldehydes and heterocycles explains the transition from a fresh, vibrant profile to the profound stale aroma that defines premium aged Ripened pu-erh tea.

### 2.4. Temporal Evolution Patterns of Volatile Metabolites via K-Means Clustering

**Figure 5:**
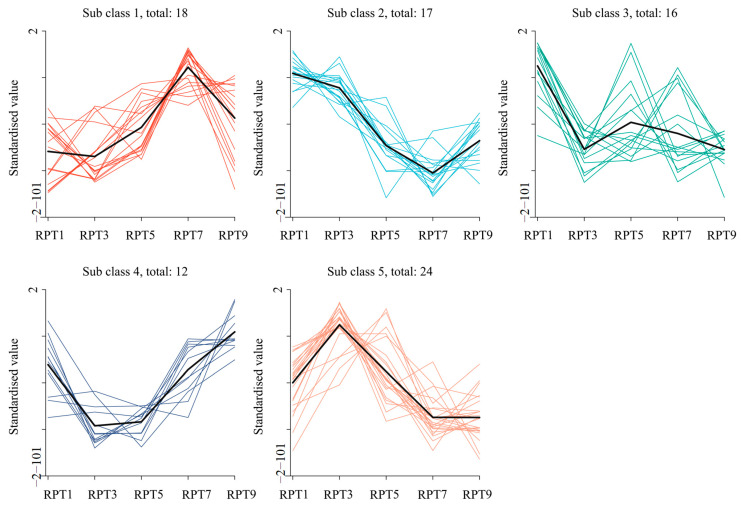
Changes in volatile compounds of RPT under different storage times. The black solid line in the figure represents the overall variation of flavor compounds in this cluster over storage time.

To decipher the kinetic trajectories of the 89 identified differential volatile organic compounds (VOCs) over the 9-year storage period, K-means clustering was performed, partitioning the metabolites into five distinct temporal patterns (Figure 5; Table S3). Based on their impact on flavor maturation and their high discriminatory power in OPLS-DA models, the accumulation profiles (Sub-classes 1 and 4) and attenuation profiles (Sub-classes 2 and 5) were prioritized for detailed investigation.

The accumulation profiles, particularly Sub-class 4 (12 VOCs) and Sub-class 1 (18 VOCs), represent the chemical foundation of maturation, characterized by a progressive enrichment of high-VIP markers that culminate in the long-term aged samples. Within these clusters, short-chain fatty acids (SCFAs) including valeric acid (VIP = 1.289) and butyric acid (VIP = 1.287) exhibited monotonic upward trends, reinforcing the mellow acidic backbone of the tea [31]. This enrichment was accompanied by the rise of critical storage markers such as *β*-ionone, which contributes to woody nuances [32], and specific methoxybenzenes, including 1,2,3-trimethoxybenzene, 3,4-dimethoxytoluene, and 2,4,6-trimethoxyacetophenone, recognized as primary constituents of the stale aroma [28]. The steady increase of these methoxybenzenes, likely formed via microbial O-methylation, serves as a vital factor driving the intensification of the stale aroma attribute [6]. These chemical trajectories are highly consistent with the sensory evolution observed in Table S1, where the sweetness (increasing from 6.17 to 7.50) and thickness (increasing from 6.17 to 7.00) scores reached their maximum in RPT9, effectively bridging the accumulation of Maillard-derived heterocyclic compounds like furfural (VIP = 1.285) and pyrrole-2-carboxaldehyde (VIP = 1.288) with the refinement of the tea’s palate. Conversely, the attenuation profiles (Sub-classes 2 and 5) comprise VOCs that predominate in the early phases before undergoing significant degradation. Sub-class 2 (17 VOCs) displays a systematic decline throughout the aging period, involving compounds that contribute to the harsh or raw characteristics of newly produced RPT. This group includes specific hydrocarbons and esters whose dissipation facilitates the transition from a pungent profile to a more balanced one. Sub-class 5 (24 VOCs), which peaks at RPT3 before progressively declining, is rich in transitional markers such as hexanal, responsible for grassy notes [19], like *α*-cyclogeraniol acetate (VIP = 1.266) and nerol (VIP = 1.263). The systematic attenuation observed in these sub-clusters aligns with the marked reduction in sourness (from 3.17 to 2.33) and bitterness (from 2.5 to 1.33) scores observed in our sensory evaluation (Table S1). This dissipation of volatile off-notes significantly refines the olfactory profile, leading to a more pure and normal flavor. In summary, the olfactory evolution of RPT is governed by a dual mechanism: the progressive accumulation of stale aroma contributors (Sub-classes 1 and 4) and the simultaneous attenuation of early-stage volatile fractions (Sub-classes 2 and 5). This chemical interplay is further supported by the quantitative volatile dynamics identified by Wang et al. [14]. provides a robust molecular explanation for the sensory transition toward the premium sweet and mellow profile characteristic of long-term aged Pu-erh tea.

### 2.5. Quantitative Evaluation of Aroma Contribution via ROAV Analysis

To further quantify the functional contribution of specific volatile markers to the overall aroma profile during aging, the relative odor activity values (ROAV) were calculated for 89 key VOCs with VIP > 1. Among these, eight compounds were identified as critical odorants with ROAV > 1 (Table 3). These high-activity substances encompass key aromatic notes, including stale, woody, toasted, sweet, grassy, roasted, and fruity nuances. Their dynamic fluctuations over the 9-year storage period provide a quantitative interpretation of the aging mechanism from the perspective of odor potency. *β*-Ionone exhibited the most dramatic shift in ROAV, surging from 35.48 at RPT1 to 244.62 at RPT5, and maintaining a high level of 212.11 at RPT9. This trend is entirely consistent with its accumulation pattern in K-means Sub-class 4, quantitatively confirming the absolute dominance of *β*-ionone as the core contributor of floral and woody notes to the total aroma activity of aged Ripened pu-erh Tea (RPT). Notably, although isovaleraldehyde maintained high ROAVs throughout all storage stages (75.97–95.38), its status evolved from a primary contributor comparable to *β*-ionone in the early stages to a secondary role. The ROAV ratio of isovaleraldehyde to *β*-ionone plummeted from 2.5 at RPT1 to 0.36 at RPT9. This reversal in relative contribution aligns closely with the sensory transition where the initial roasted impression is gradually superseded by the matured stale aroma. Among the stale aroma markers, 1,2,3-trimethoxybenzene showed a monotonic upward trend in ROAV, rising steadily from 11.59 (RPT1) to 15.79 (RPT9). Conversely, the ROAV of 1,2,3,4-tetramethoxybenzene fluctuated (2.06 to 5.24 to 2.00) without significant net accumulation. This disparity suggests that even among methoxybenzene-type stale components, the dynamic contribution to the aging aroma is structure-dependent; the sustained increase in the activity of 1,2,3-trimethoxybenzene likely serves as a vital molecular foundation for the intensifying stale aroma attribute. In sharp contrast to the accumulation-type compounds, the ROAVs of hexanal and propionaldehyde, which are associated with grassy and fruity characteristics, exhibited a downward trend. The ROAV of hexanal decreased continuously from 1.08 (RPT1) to 0.56 (RPT9), while propionaldehyde fluctuated downward from 1.02 to 0.74, indicating a gradual weakening of their direct odor activity. Simultaneously, the overall activity of dihydroactinidiolide (musty/sweet, ROAV decreasing from 2.71 to 1.90) and 2-methylfuran (toasted, fluctuating between 1.36–1.64 without increase) failed to escalate. The attenuation or stagnation of these active substances echoes the kinetics of Sub-classes 2 and 5, explaining the dissipation of raw notes such as grassy and fruity aromas, as well as the reduction in sourness and bitterness scores from the perspective of odor activity (Table S1).

In summary, ROAV analysis provides quantitative evidence that confirms and deepens the dual kinetic mechanism revealed by K-means clustering: the accumulation of high-activity substances represented by *β*-ionone and 1,2,3-trimethoxybenzene drives the aromatic shift toward stale, woody, and floral notes. Simultaneously, the relative weakening of isovaleraldehyde and the attenuation of raw-tea markers like hexanal and propionaldehyde contribute to a more pure and harmonious aroma profile. This rebalancing of odor activity values provides critical quantitative support for the formation of the sweet and mellow flavor characteristic of long-term aged RPT.

## 3. Materials and Methods

### 3.1. Chemical Standards

Reference standards (>98% purity) for 18 compounds, gallic acid (GA), ellagic acid (EA), caffeine (CA), quercetin (QU), luteolin (LU), kaempferol (KAE), myricetin (MY), theophylline (THEO), rutin (RUT), taxifolin (TAX), (+)-catechin (C), (−)-epicatechin (EC), (−)-epigallocatechin (EGC), (−)-epicatechin 3-O-gallate (ECG), (−)-epigallocatechin 3-O-gallate (EGCG), (−)-gallocatechin (GC), (−)-gallocatechin gallate (GCG) and (−)-catechin gallate (CG) of HPLC grade were purchased from Manster Biotechnology Co., Ltd. (Chengdu, China). HPLC-grade acetonitrile and methanol were from Beijing Mirida Technology Co., Ltd., (Beijing, China).

### 3.2. RPT Sample Collection

The experimental materials consisted of “Laotongzhi 9978” ripened pu-erh tea cakes (357 g per cake) produced by Anning Haiwan Tea Industry Co., Ltd. (Kunming, China). This product is manufactured using a standardized recipe designed for batch-to-batch consistency. Samples were produced in five different calendar years using raw materials sourced from Menghai, Xishuangbanna, Yunnan and processed under the same standardized fermentation and compression protocol. For each of the five storage years (1, 3, 5, 7, and 9 years post-fermentation), three independent tea cakes were collected (15 cakes in total) and labeled as RPT1, RPT3, RPT5, RPT7, and RPT9, respectively. Each cake was analyzed individually for all chemical and sensory determinations, providing three biological replicates per storage year.

All RPT samples were stored in a professional dedicated tea warehouse located in Kunming, Yunnan Province, China. This region is characterized by a typical dry storage environment, with an annual average temperature generally maintained between 15 °C and 25 °C and a relative humidity (RH) ranging from 50% to 70%. To ensure consistency and prevent microbial contamination or exogenous odors, the tea cakes were kept in their original double-layer cotton paper packaging, bundled in bamboo husks, and housed in standardized cardboard boxes. The warehouse featured natural ventilation with controlled air circulation and was strictly protected from direct sunlight and environmental pollutants.

We acknowledge that, although three independent cakes per storage year were used to provide biological replicates and reduce intra-year variability, all samples originated from a single commercial product and production recipe. This design was deliberately chosen to minimize confounding variables (e.g., raw material provenance, processing batch effects) and thereby strengthen our ability to attribute observed chemical-sensory differences primarily to storage duration. However, this approach necessarily limits generalizability: the observed aging trajectories are specific to this product and may not fully represent the diversity of RPT products.

### 3.3. Sensory Evaluation

The sensory evaluation was conducted in accordance with the Chinese National Standard (GB/T 23776-2018) [33]. To ensure the statistical robustness and representativeness of the findings, sensory profiling was performed independently for each of the three biological replicates (*n* = 3) per storage year. For each replicate, a panel of nine trained sensory testers (five males and four females, aged 24 to 48 years) evaluated the samples. First, the appearance of the dry tea leaves was graded on bamboo dividing trays. Subsequently, the tea infusion was prepared by adding 3 g of dry tea leaves to 150 mL of boiling water for a 5-min extraction. The infusions were labeled with randomized three-digit codes and presented in white porcelain bowls in individual sensory booths. The liquor color, aroma (evaluated via a three-stage sniffing method), and flavor (at 55 ± 2 °C) were assessed in a fixed sequence. In addition, specific taste profiles (bitterness, sweetness, sourness, and umami) and mouthfeel (astringency and thickness) were quantified using modified Quantitative Descriptive Analysis (QDA) following the established framework [34]. The intensity level of each attribute was scored on a numerical scale ranging from 0 (undetectable) to 10 (very strong). The final scores for each aging stage were derived from the mean of the assessments across the three independent biological replicates.

### 3.4. Instrumental Color Measurement

To provide a quantitative assessment of the liquor color, the chromaticity parameters (L*, a*, and b*) of the tea infusions were measured using a chromameter YS6060 (San En Shi Technology Co., Ltd., Shenzhen, China). Prior to measurement, the infusions were filtered through a 0.45 μm membrane. The measurements were conducted in transmittance mode using a 10 mm path-length quartz cuvette under standard illuminant D65 and a 10° observer angle. Measurements were performed independently for each of the three biological replicates per storage year to account for inter-cake variability.

### 3.5. Chemical Composition Analysis

#### 3.5.1. Determination of Major Chemical Compositions

The total contents of water extracts (WE), tea polyphenols (TP), free amino acids (FAA), soluble sugars (SS), theaflavins (TF), thearubigins (TR), and theabrownins (TB) in tea infusions were determined as described previously [35]. To ensure the findings were representative of the storage gradient, all chemical analyses were performed independently for each of the three biological replicates (*n* = 3) per storage year.

Water extracts (WE): Briefly, 1.0 g of ground tea (40-mesh) was extracted with 200 mL of boiling distilled water in a boiling water bath for 45 min. The residue was filtered, dried at 120 ± 2 °C to constant weight, and weighed.

Free amino acids (FAA): The total FAA content was measured using the ninhydrin colorimetric method with glutamic acid as the standard. Absorbance was recorded at 570 nm using a UV-Vis spectrophotometer (model and manufacturer, if available).

Tea polyphenols (TP): TP content was determined by the Folin-Ciocalteu (FC) method. Samples were extracted with 70% methanol at 70 °C, and the absorbance was measured at 765 nm using a UV-Vis spectrophotometer. Results were expressed as gallic acid equivalents (GAE).

Soluble sugars (SS): SS content was analyzed using the anthrone-sulfuric acid method with glucose as the standard. Absorbance was measured at 620 nm.

Tea pigments (TF, TR, and TB): Tea pigments were quantified using the systematic solvent extraction method. The infusions were sequentially partitioned with ethyl acetate and n-butanol. The absorbance of the resulting organic fractions was measured at 380 nm and 278 nm using a UV-Vis spectrophotometer, and the concentrations of TF, TR, and TB were calculated accordingly.

#### 3.5.2. Quantification of Phenolic Compounds and Alkaloids by HPLC

The quantification of individual phenolic compounds [36], including GA, CA, EA, QU, LU, KA, MY, C, EC, EGC, ECG, EGCG, GC, GCG, and CG in tea sample, was determined using an Agilent 1200 series HPLC system equipped with a Poroshell 120 EC-C18 column 4.6 × 100 mm, 2.7 μm. Mobile Phase: Solvent A (0.261% phosphoric acid and 5% acetonitrile) and Solvent B (0.261% phosphoric acid and 80% acetonitrile). Gradient Program: 0–21 min, 10–20% B; 21–22 min, 20–100% B; 22–26 min, 100% B; 26–26.5 min, 100–10% B. The program stopped at 27 min with a 5 min post-run equilibration. Conditions: Flow rate: 1.0 mL/min; Column temperature: 40 °C. Detection: The wavelength was set at 280 nm from 0–16 min and switched to 360 nm from 16–30 min. Each of the three independent biological replicates was analyzed separately to capture the chemical variance across the nine-year storage gradient.

### 3.6. HS-GC-MS Conditions

Volatile compounds were extracted and analyzed using HS-GC-MS by Shanghai Meiji Biomedical Technology Co., Ltd. (Shanghai, China). The GC-MS analysis was performed on an Agilent 8890 gas chromatography (Agilent, Santa Clara, CA, USA) coupled with an Agilent 7000D mass selective detector (Agilent, Santa Clara, CA, USA), which was equipped with an electron impact (EI) ionization source and worked in full scan mode. A VF-WAXms quartz capillary column (25 m × 0.25 mm × 0.2 µm) sourced from Agilent Technologies (Santa Clara, CA, USA) was used for separation. Headspace extraction was performed by an Agilent 7697A autosampler.

HS parameters were as follows: 3 g of the RPT samples was accurately weighed and added into 20 mL headspace vials, and immediately sealed, and then heated at 130 °C with an equilibration time of 30 min. The temperatures of the quantitative ring and the transmission line were maintained at 150 and 170 °C, respectively. The samples were injected into the GC-MS system in split mode for analysis with the injection time of 0.5 min and the injection volume of 1.0 µL.

GC parameters were as follows: The RPT samples were separated on a VF-WAXms capillary column (25 m × 0.25 mm × 0.2 µm), the split ratio was set to 10:1, the inlet temperature was 180 °C, and the carrier gas was helium (99.999% pure) with a constant flow rate of 2.0 mL/min. The oven temperature program started from 40 °C, was held for 2 min, and increased to 100 °C at a rate of 5 °C/min, then heated to 230 °C at a rate of 15 °C/min, remaining there for 5 min. After that, the column temperature was maintained at 230 °C for 2 min. The total run time of the GC program was 35 min. To evaluate the stability of the analytical system during the run-on process, all the samples along with Quality controls (QCs) were injected into the GC-MS. During instrument testing, a QC sample was inserted every 5–15 samples.

MS parameters were as follows: the inert electron impact (EI) energy was 70 eV. Using the full scan mode over the mass range m/z 30 to 1000 at 3.2 scan/s. Temperatures of the ion source, the transmission line, and the quadrupole were set at 230 °C, 310 °C, and 150 °C.

The pretreatment of GC/MS raw data was performed by MassHunter workstation Quantitative Analysis (version v10.0.707.0) software, and a three-dimensional data matrix in CSV format was exported. The information in this three-dimensional matrix included: sample information, metabolite name, and mass spectral response intensity. Internal standard peaks, as well as any known false positive peaks (including noise, column bleed, and derivatized reagent peaks), were removed from the data matrix, deredundant, and peak pooled. At the same time, the metabolites were identified by searching databases, and the main databases were public databases such as NIST (version 2017), Fiehn (version 2013), and MS-DIAL (version 2021). Quantification was performed using the peak area normalization method, with the results expressed as the relative content (%) of each compound. To ensure data reliability across the 9-year gradient, all samples were analyzed in a single continuous batch under identical extraction and instrumental parameters. The results reported for each storage year represent the mean values derived from the three independent biological replicates.

It should be noted that the headspace extraction in this study was performed at 130 °C, which is higher than the 60–80 °C range commonly employed in SPME-based tea aroma studies. While this elevated temperature was deliberately chosen to enhance the volatilization of semi-volatile compounds such as methoxybenzenes that are critical to aged RPT aroma, it may also promote thermal artifacts, including the degradation of thermolabile compounds and in-vial Maillard reactions. Consequently, the relative abundance of heat-sensitive compound classes, particularly furans and pyrrole derivatives, should be interpreted with caution, as they may partly reflect extraction-induced reactions rather than the native volatile composition of the tea. Future studies employing complementary low-temperature SPME methods would be valuable for independent validation.

### 3.7. Statistical Analysis

All experimental data were expressed as mean ± standard deviation (SD) of three independent biological replicates (*n* = 3). One-way analysis of variance (ANOVA) followed by Duncan’s multiple range test was performed using SPSS (version 26.0, IBM Corp., Armonk, NY, USA) to compare chemical components, chromaticity parameters, and QDA sensory scores among tea samples of different storage years, with statistical significance set at *p* < 0.05.

Pearson correlation analysis between QDA sensory attributes and characteristic chemical components was conducted using the Metware Cloud, a free online platform for data analysis (https://cloud.metware.cn, accessed on 13 March 2026, Wuhan, China). The correlation matrix was visualized as a heatmap using TBtools software (Toolbox for Biologists; Version 1.082, Guangzhou, China).

For volatile compound data, multivariate statistical analyses were performed on the Majorbio Cloud Platform (https://www.majorbio.com/tools accessed on 13 March 2026, Shanghai, China). Principal Component Analysis (PCA) was conducted to visualize the overall divergence of volatile profiles among storage years. Orthogonal Partial Least Squares Discriminant Analysis (OPLS-DA) was used to identify volatile compounds discriminating between RPT1 and each subsequent storage stage (RPT3, RPT5, RPT7, and RPT9). Model robustness was validated by permutation testing (200 permutations), with R^2^Y and Q^2^ values used to assess goodness-of-fit and predictive ability, respectively. Variable Importance in Projection (VIP) scores were extracted from the OPLS-DA models, and compounds with VIP > 1.0 and *p* < 0.05 were considered significantly discriminatory. K-means clustering was performed on the Majorbio Cloud Platform to categorize the temporal evolution patterns of differential volatile compounds across the nine-year storage period.

Radar plots of QDA scores were generated using Orange (version 3.0, University of Ljubljana, Slovenia). Bar charts of chromaticity values (L*, a*, b*) were plotted using GraphPad Prism (version 9.0, GraphPad Software, San Diego, CA, USA).

## 4. Conclusions

This study characterized the quality dynamics of a standardized commercial RPT product across a 9-year storage gradient. Organoleptically, RPT samples progressed from a heavy and mellow mouthfeel with an approaching pure aroma in the early stages toward a sweet and mellow character with a pure and normal stale aroma in the later stages. Visually, the infusion evolved from reddish-brown to a deeper, heavy reddish hue, quantitatively corroborated by significant increases in redness (*a*) and yellowness (*b*) indices (*p* < 0.05). Chemically, this sensory maturation was driven by the significant degradation of astringent esterified catechins (e.g., EGCG and ECG, *p* < 0.05) alongside the steady polymerization of infusion pigments, which reduced harshness and improved palate thickness. The aroma evolution was characterized by a dual volatile mechanism: the progressive enrichment and increasing ROAV of stale aroma contributors (1,2,3-trimethoxybenzene and *β*-ionone) and the simultaneous attenuation of short-chain acids, aldehydes, and furan derivatives. ROAV analysis provided quantitative olfactory evidence confirming that the relative activity of *β*-ionone and 1,2,3-trimethoxybenzene increasingly dominates the aroma profile, while the contributions of grassy and roasted notes progressively weaken. Collectively, these results suggest that, within the context of this standardized commercial product, the maturation of RPT is characterized by the intensification of characteristic stale aroma compounds and the reduction of specific aldehydes and acids, providing objective chemical indicators for the quality assessment of aged tea. It should be noted that the cross-sectional design of this study—using five independent production batches rather than a single batch followed longitudinally—constitutes a limitation, and the identified markers should be regarded as product-specific candidates requiring validation across a broader range of products and through true longitudinal studies.

## Figures and Tables

**Table 1:** Descriptive sensory evaluation of RPT samples across a nine-year storage gradient.

Storage Year	Appearance	Infusion Color	Aroma	Taste	Infused Leaves
RPT1	Reddish brown, slightly tippy, more bloom	Reddish brown, bright	Stale aroma, woody aroma, approach pure	Heavy and mellow	Reddish brown, more bloom, soft
RPT3	Reddish brown, slightly tippy, approach bloom	Reddish brown, bright	Stale aroma, woody aroma, approach pure	Mellow and thick	Reddish brown, more bloom, soft
RPT5	Reddish brown, slightly tippy, more bloom	Reddish, bright	Stale aroma, woody aroma, pure and normal	Mellow	Reddish brown, more bloom, soft
RPT7	Reddish brown, slightly tippy, more bloom	Heavy reddish, bright	Stale aroma, woody aroma, pure and normal	Mellow	Reddish brown, more bloom, soft
RPT9	Reddish brown, slightly tippy, more bloom	Heavy reddish, bright	Stale aroma, woody aroma, pure and normal	Sweet and mellow	Reddish brown, more bloom, soft

**Table 2:** HPLC-based profiling of dynamic changes in characteristic components of RPT during storage.

Sample	RPT1	RPT3	RPT5	RPT7	RPT9
TB (%)	14.04 ± 1.13 ^a^	13.66 ± 0.80 ^a^	13.66 ± 0.10 ^a^	13.91 ± 0.20 ^a^	14.24 ± 0.33 ^a^
TR (%)	2.17 ± 1.11 ^b^	1.46 ± 0.20 ^b^	1.38 ± 0.10 ^b^	6.22 ± 0.50 ^a^	6.10 ± 0.30 ^a^
TP (%)	7.71 ± 0.84 ^bc^	9.54 ± 0.10 ^a^	8.87 ± 0.90 ^ab^	6.55 ± 0.40 ^c^	4.41 ± 0.80 ^d^
FAA (%)	0.55 ± 0.03 ^c^	0.62 ± 0.02 ^b^	0.69 ± 0.04 ^a^	0.40 ± 0.01 ^d^	0.40 ± 0.02 ^d^
SS (%)	4.36 ± 0.20 ^c^	4.78 ± 0.61 ^c^	8.37 ± 0.61 ^a^	6.87 ± 0.13 ^b^	4.70 ± 0.71 ^c^
WE (%)	34.17 ± 1.62 ^a^	34.37 ± 0.43 ^a^	47.54 ± 14.74 ^a^	33.60 ± 2.91 ^a^	35.88 ± 0.33 ^a^
TF (%)	0.21 ± 0.03 ^c^	0.20 ± 0.01 ^c^	0.61 ± 0.02 ^a^	0.34 ± 0.05 ^b^	0.37 ± 0.03 ^b^
THEO (mg/g)	0.12 ± 0.01 ^a^	0.08 ± 0.01 ^b^	0.05 ± 0.01 ^c^	0.06 ± 0.01 ^c^	0.04 ± 0.01 ^c^
EC (mg/g)	0.86 ± 0.10 ^b^	1.25 ± 0.10 ^a^	0.59 ± 0.30 ^b^	0.59 ± 0.05 ^b^	0.15 ± 0.01 ^c^
GCG (mg/g)	0.37 ± 0.10 ^b^	0.46 ± 0.02 ^a^	0.37 ± 0.02 ^b^	0.26 ± 0.02 ^c^	0.27 ± 0.02 ^c^
EGCG (mg/g)	0.43 ± 0.10 ^b^	0.82 ± 0.02 ^a^	0.37 ± 0.04 ^b^	0.28 ± 0.03 ^c^	0.27 ± 0.03 ^c^
ECG (mg/g)	0.52 ± 0.01 ^b^	1.00 ± 0.03 ^a^	0.57 ± 0.02 ^b^	0.39 ± 0.03 ^c^	0.33 ± 0.03 ^d^
MY (mg/g)	0.04 ± 0.01 ^a^	0.05 ± 0.01 ^a^	0.04 ± 0.01 ^a^	0.04 ± 0.01 ^a^	0.04 ± 0.01 ^a^
EGC (mg/g)	0.88 ± 0.10 ^b^	1.26 ± 0.03 ^a^	0.54 ± 0.04 ^c^	0.39 ± 0.06 ^d^	0.69 ± 0.07 ^c^
C (mg/g)	0.92 ± 0.10 ^b^	1.35 ± 0.05 ^a^	0.15 ± 0.01 ^c^	0.10 ± 0.01 ^c^	0.13 ± 0.01 ^c^
TAX (mg/g)	0.08 ± 0.01 ^a^	0.09 ± 0.10 ^a^	0.05 ± 0.01 ^a^	0.05 ± 0.01 ^a^	0.05 ± 0.01 ^a^
CG (mg/g)	0.11 ± 0.01 ^b^	0.07 ± 0.01 ^d^	0.09 ± 0.01 ^c^	0.13 ± 0.01 ^a^	0.06 ± 0.01 ^d^
QU (mg/g)	0.17 ± 0.01 ^b^	0.17 ± 0.01 ^b^	0.17 ± 0.01 ^b^	0.21 ± 0.01 ^a^	0.16 ± 0.01 ^b^
CA (mg/g)	33.72 ± 2.10 ^b^	36.07 ± 0.50 ^ab^	36.52 ± 0.41 ^a^	37.31 ± 0.60 ^a^	35.72 ± 1.01 ^ab^
EA (mg/g)	1.72 ± 0.03 ^b^	2.14 ± 0.11 ^a^	2.13 ± 0.10 ^a^	2.26 ± 0.06 ^a^	2.17 ± 0.31 ^a^
KA (mg/g)	0.08 ± 0.01 ^ab^	0.08 ± 0.01 ^ab^	0.09 ± 0.01 ^a^	0.08 ± 0.01 ^b^	0.08 ± 0.01 ^ab^
GC (mg/g)	25.52 ± 2.94 ^a^	28.42 ± 0.72 ^a^	28.56 ± 0.60 ^a^	27.20 ± 0.80 ^a^	26.74 ± 1.03 ^a^
RUT (mg/g)	0.85 ± 0.01 ^b^	1.08 ± 0.02 ^ab^	1.20 ± 0.05 ^a^	0.96 ± 0.30 ^ab^	0.92 ± 0.07 ^ab^
GA (mg/g)	8.34 ± 0.60 ^cd^	10.71 ± 0.28 ^b^	13.40 ± 0.21 ^a^	7.62 ± 1.02 ^d^	9.48 ± 0.22 ^bc^
LU (mg/g)	0.03 ± 0.01 ^b^	0.04 ± 0.01 ^b^	0.04 ± 0.01 ^a^	0.03 ± 0.01 ^b^	0.03 ± 0.01 ^b^

Note: Values are presented as Mean ± SD (*n* = 3). Different lowercase superscript letters (a, b, c...) within the same column indicate significant differences at *p* < 0.05 based on one-way ANOVA followed by Duncan’s multiple range test. Abbreviations: gallic acid (GA), ellagic acid (EA), caffeine (CA), quercetin (QU), luteolin (LU), kaempferol (KA), myricetin (MY), theophylline (THEO), rutin (RUT), taxifolin (TAX), (+)-catechin (C), (−)-epicatechin (EC), (−)-epigallocatechin (EGC), (−)-epicatechin 3-O-gallate (ECG), (−)-epigallocatechin 3-O-gallate (EGCG), (−)-gallocatechin (GC), (−)-gallocatechin gallate (GCG) and (−)-catechin gallate (CG), water extracts (WE), tea polyphenols (TP), free amino acids (FAA), soluble sugars (SS), theaflavins (TF), thearubigins (TR), and theabrownins (TB).

**Table 3:** Key aroma-active compounds and their ROAVs in Ripened Pu-erh Tea (RPT) during a 9-year aging period.

Metab ID	Metabolite	OT (ug/kg)	Odor Quality	Class	CAS ID	Formula	Roav				
							RPT1	RPT3	RPT5	RPT7	RPT9
metab_6	1,2,3,4-Tetramethoxybenzene	0.64	stale, woody	Benzenes	21450-56-6	C_10_H_14_O_4_	2.06 ± 0.16 ^c^	5.24 ± 0.07 ^a^	2.53 ± 0.11 ^b^	1.18 ± 0.10 ^d^	2.00 ± 0.05 ^c^
metab_7	1,2,3-Trimethoxybenzene	0.75	stale, woody	Benzenes	634-36-6	C_9_H_12_O_3_	11.59 ± 0.60 ^c^	12.73 ± 0.35 ^c^	14.45 ± 0.87 ^b^	16.26 ± 1.49 ^a^	15.79 ± 0.10 ^ab^
metab_111	2-Methylfuran	11	Toasted	Heterocyclic compound	534-22-5	C_5_H_6_O	1.51 ± 0.07 ^b^	1.64 ± 0.04 ^a^	1.56 ± 0.06 ^ab^	1.37 ± 0.07 ^c^	1.36 ± 0.04 ^c^
metab_26	Dihydroactinidiolide	2.1	woody, sweet	Esters	17092-92-1	C_11_H_16_O_2_	2.71 ± 0.09 ^a^	2.69 ± 0.06 ^a^	2.20 ± 0.10 ^b^	1.79 ± 0.08 ^c^	1.90 ± 0.07 ^c^
metab_122	Hexanal	2.4	Grassy	Aldehydes	66-25-1	C_6_H_12_O	1.08 ± 0.04 ^b^	1.19 ± 0.06 ^a^	0.88 ± 0.02 ^c^	0.57 ± 0.04 ^d^	0.56 ± 0.03 ^d^
metab_83	Isovaleraldehyde	0.25	Roasted	Aldehydes	590-86-3	C_5_H_10_O	88.80 ± 10.12 ^ab^	92.11 ± 1.20 ^ab^	95.38 ± 16.98 ^a^	87.88 ± 2.38 ^ab^	75.97 ± 3.43 ^b^
metab_156	Propionaldehyde	7	Fruity	Aldehydes	123-38-6	C_3_H_6_O	1.02 ± 0.02 ^ab^	1.23 ± 0.03 ^a^	0.99 ± 0.08 ^ab^	1.09 ± 0.31 ^a^	0.74 ± 0.08 ^b^
metab_51	*β*-Ionone	0.021	Floral, woody	Ketones	14901-07-6	C_13_H_20_O	35.48 ± 1.32 ^d^	44.97 ± 0.23 ^d^	244.62 ± 6.51 ^a^	189.68 ± 15.83 ^c^	212.11 ± 8.36 ^b^

Different lowercase superscript letters (a, b, c...) within the same column indicate significant differences at *p* < 0.05 based on one-way ANOVA followed by Duncan’s multiple range test.

## Data Availability

The datasets supporting the findings of this study, including non-volatile chemical compositions, VOC profiles, and sensory evaluation results, are available within the article and its Supplementary Material files (Tables S1–S3).

## Supplementary Materials

The following supporting information can be downloaded at: https://www.mdpi.com/article/10.3390/molecules31111937/s1, Figure S1. OPLS-DA analysis of volatile metabolites comparing RPT3, 5, 7, 9 VS RPT1. (A) RPT3 vs. RPT1; (B) RPT5 vs. RPT1; (C) RPT7 vs. RPT1; (D) RPT9 vs. RPT1; Table S1. Sensory attribute intensities of RPT samples across a 9-year storage gradient determined by the QDA scores; Table S2. Instrumental chromaticity values (*L**, *a**, *b**) and statistical significance analysis of RPT liquor color; Table S3. K-Means clustering table of differential volatile compounds in RPT.
