# Supplementary material for: Metabolomic and Sensory Insights into the Aging Mechanism of Ripened Pu-Erh Tea over Nine Years
Source: Molecules. 2026 Jun 3;31(11):1937. doi: 10.3390/molecules31111937 (PMC13258448; doi:10.3390/molecules31111937)
Supplement: Supplementary file 1 [file molecules-31-01937-s001.zip › molecules-4305326-supplementary.pdf]

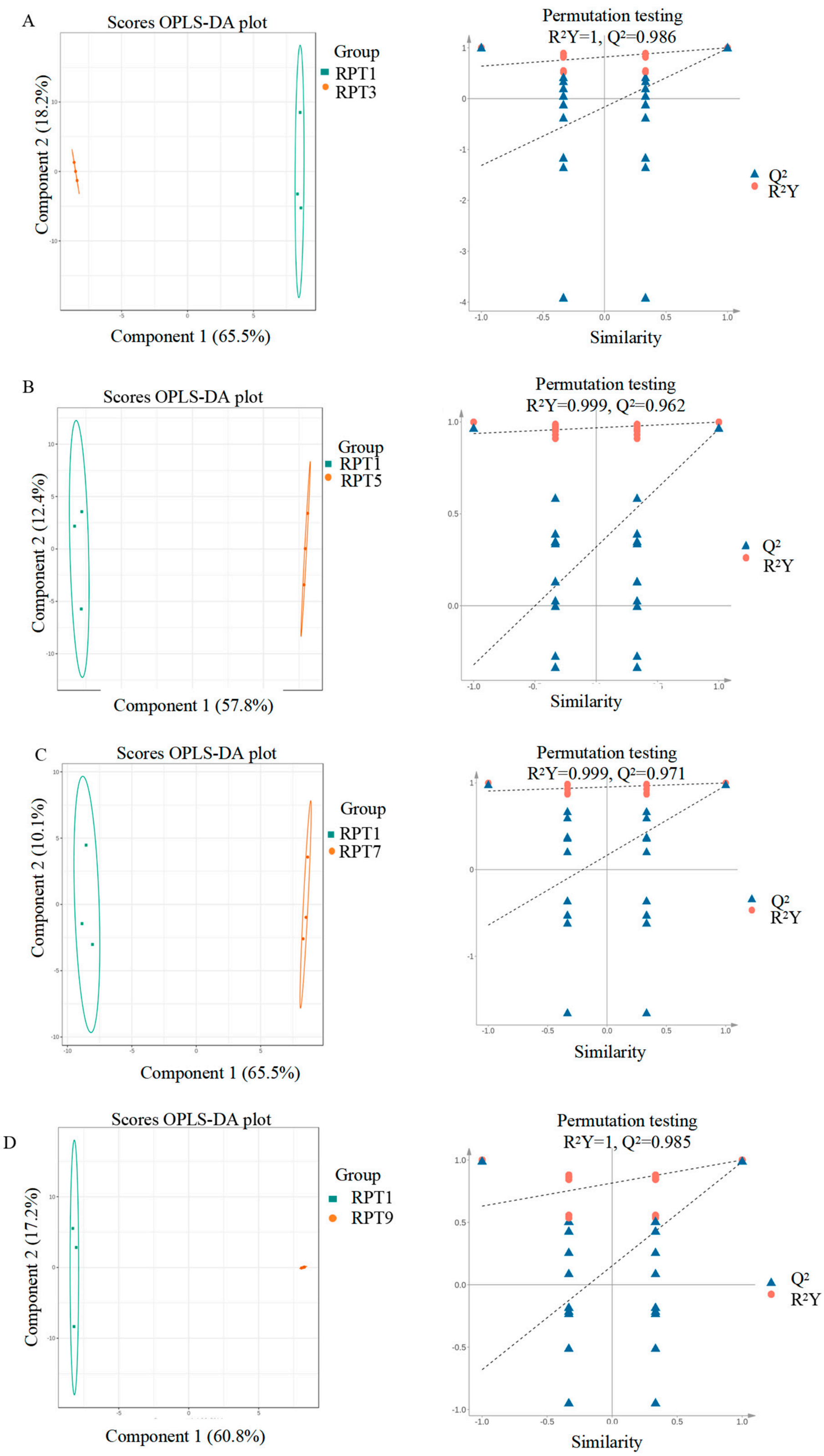

Figure S1. OPLS-DA analysis of volatile metabolites comparing RPT3, 5, 7, 9 VS RPT1. **(A)** RPT3 vs RPT1; **(B)** RPT5 vs RPT1; **(C)** RPT7 vs RPT1; **(D)** RPT9 vs RPT1.

Table S1. Sensory attribute intensities of RPT samples across a 9-year storage gradient determined by the QDA scores.

	Bitterness	Umami	Astringency	Sourness	Thickness	Sweetness
RPT1	2.5±0.5[a]	1.5±0.5[a]	3.17±0.29[a]	3.17±0.29[a]	6.17±0.29[b]	6.17±0.29[d]
RPT3	2.07±0.12[ab]	1.17±0.29[a]	2.5±0.5[ab]	2.5±0.5[b]	6.17±0.29[b]	6.33±0.29[cd]
RPT5	1.5±0.5[bc]	1.17±0.29[a]	2.5±0.5[ab]	2.17±0.29[b]	6.33±0.29[b]	6.83±0.29[bc]
RPT7	1.33±0.29[c]	1.5±0[a]	1.83±0.29[b]	2.33±0.29[b]	6.67±0.29[ab]	7.17±0.29[ab]
RPT9	1.17±0.29[c]	1.17±0.29[a]	1.67±0.58[b]	1.17±0.29[c]	7.17±0.29[a]	7.67±0.29[a]

Note: Values are expressed as mean ± SD. Different lowercase letters within the same column indicate significant differences according to Duncan's multiple range test ($p < 0.05$).

Table S2. Instrumental chromaticity values (L^*, a^*, b^*) and statistical significance analysis of RPT liquor color.

	L^*	a^*	b^*
RPT1	69.58±0.02[d]	22.6±0.09[b]	67.19±0.28[b]
RPT3	71.14±0.03[c]	20.47±0.04[c]	64.42±0.19[c]
RPT5	74.61±0.03[a]	15.63±0.05[e]	56.21±0.11[e]
RPT7	72.67±0.01[b]	17.7±0.03[d]	62.85±0.05[d]
RPT9	67.4±0.01[e]	23.12±0.02[a]	68.95±0.06[a]

Note: Values are expressed as mean ± SD. Different lowercase letters within the same column indicate significant differences according to Duncan's multiple range test ($p < 0.05$).

Table S3. K-Means clustering table of differential volatile compounds in RPT

Subclass（K-Means）	Metab ID	Metabolite	CAS ID	Formula	Class	Relative content（%）														
						RPT1-1	RPT1-2	RPT1-3	RPT3-1	RPT3-2	RPT3-3	RPT5-1	RPT5-2	RPT5-3	RPT7-1	RPT7-2	RPT7-3	RPT9-1	RPT9-2	RPT9-3
1	metab_128	Methyl salicylate	119-36-8	C8H8O3	Esters	0.17936	0.15509	0.16695	0.11715	0.0876	0.10267	0.12887	0.14181	0.15047	0.21052	0.21194	0.24245	0.11995	0.13445	0.12728
1	metab_135	N-Ethylformamide	627-45-2	C3H7NO	Others	0.14647	0.18937	0.18787	0.1652	0.15727	0.16131	0.16472	0.17236	0.1784	0.24311	0.24241	0.23273	0.21105	0.19582	0.20336
1	metab_147	4-Amino-2,5-dimethylphenol	3096-71-7	C8H11NO	Phenols	0.45075	0.40073	0.38964	0.309	0.30213	0.30563	0.34744	0.3409	0.39357	0.62958	0.55765	0.67772	0.41925	0.43893	0.42919
1	metab_148	4-Amino-3-methylphenol	2835-99-6	C7H9NO	Phenols	5.75432	5.12903	6.04926	4.82396	4.13432	4.48591	5.08671	4.72792	5.46998	7.69203	6.73762	7.90328	5.40391	5.51481	5.45994
1	metab	1-Ethylpyrrole	617-92-	C6H9	Heterocyclic	0.80538	0.74937	0.88863	0.60081	0.49639	0.54963	0.74434	0.65455	0.77412	1.14446	1.10059	1.32458	0.83078	0.99862	0.91558

	_19		5	N	compound															
1	metab_28	2,3,4-Trimethyl-1H-pyrrole	3855-78-5	C7H11N	Heterocyclic compound	0.14182	0.1243	0.1623	0.07928	0.07569	0.07752	0.1052	0.1047	0.11603	0.18934	0.18627	0.25268	0.14689	0.1985	0.17297
1	metab_30	2,3-dimethylfuran	14920-89-9	C6H8O	Heterocyclic compound	4.143	5.06986	5.69	5.69156	6.415	6.04618	8.95216	7.51366	6.82949	7.02767	8.5692	7.04971	7.31463	8.14032	7.73183
1	metab_33	2',4',6'-Trimethoxyacetophenone	832-58-6	C11H14O4	Ketones	0.62415	0.50606	0.68629	0.78078	0.69982	0.74109	0.85143	0.93225	0.90653	1.01419	0.92288	0.9703	1.00086	0.95494	0.97766
1	metab_47	2-Methyltetrahydrofuran-3-one	3188-00-9	C5H8O2	Ketones	0.11601	0.09037	0.09446	0.10871	0.11394	0.11127	0.10171	0.09178	0.09019	0.12773	0.12603	0.13626	0.0898	0.09806	0.09397
1	metab_48	3,4-Dimethoxytoluene	494-99-5	C9H12O2	Benzenes	0.11325	0.15074	0.18106	0.12432	0.15125	0.13752	0.26507	0.22611	0.18102	0.19977	0.21129	0.22321	0.19185	0.29044	0.24166
1	metab_51	β-Ionone	14901-07-6	C13H20O	Ketones	0.06939	0.06809	0.21455	0.0858	0.08491	0.08536	0.47625	0.06516	0.45158	0.35484	0.39237	0.33295	0.41854	0.38681	0.40251

1	metab_55	4-Acetyl-1H-pyrrole-2-carbaldehyde	16168-92-6	C7H7NO2	Aldehydes	0.56681	0.52543	0.49976	0.42036	0.38879	0.40488	0.45536	0.41035	0.49287	0.74221	0.725	0.83455	0.54188	0.58636	0.56435
1	metab_60	4-Terpinenyl acetate	1066974	C12H20O2	Esters	0.23305	0.22023	0.20739	0.19987	0.23023	0.21475	0.21419	0.20025	0.1888	0.29118	0.2725	0.31361	0.22836	0.03727	0.13181
1	metab_63	Acetaldehyde, hydroxy-(9CI)	141-46-8	C2H4O2	Aldehydes	0.7134	0.67919	0.65406	0.87182	0.81395	0.84345	0.84019	0.78441	0.85136	0.98149	0.94523	0.92248	0.72552	0.67952	0.70228
1	metab_65	N-Ethylacetamide	625-50-3	C4H9NO	Others	0.18372	0.2171	0.21466	0.16327	0.14623	0.15492	0.18591	0.18616	0.20175	0.27867	0.29861	0.28548	0.23502	0.2243	0.2296
1	metab_7	1,2,3-Trimethoxybenzene	634-36-6	C9H12O3	Benzenes	0.80365	0.81433	0.73912	0.88689	0.83903	0.86343	1.03663	0.91892	0.98261	1.10969	1.19871	0.99755	1.07757	1.06383	1.07063
1	metab_86	Caffeine	114303-55-8	C8H10N4O2	Others	6.02623	5.19457	6.34512	7.75957	6.34602	7.06668	7.76288	8.45867	8.59493	9.82525	8.79815	8.88026	9.3663	8.874	9.11755
2	me	2-Acetylfuran	119	C6	Hetero	0.34	0.32	0.36	0.34	0.33	0.33	0.29	0.27	0.30	0.30	0.27	0.30	0.25	0.27	0.26

	tab_106		2-62-7	H6O2	cyclic compound	79	608	237	614	066	855	324	556	227	524	288	976	09	094	102
2	metab_107	Acetosyringone	2478-38-8	C10H12O4	Ketones	0.21275	0.24511	0.25713	0.17388	0.22882	0.20081	0.21762	0.23248	0.18566	0.06358	0.06056	0.06602	0.14921	0.13928	0.14419
2	metab_113	Furfuryl Alcohol	98-00-0	C5H6O2	Alcohols	5.4347	5.35291	5.40471	4.64308	5.01913	4.82741	4.43466	4.63053	4.61803	4.30276	4.15268	4.5368	4.64925	4.78579	4.71824
2	metab_123	Hexaric Acid	142-62-1	C6H12O2	Acids	0.70593	0.76561	0.8004	0.58929	0.5105	0.55067	0.41388	0.41916	0.47028	0.259	0.23473	0.2535	0.22973	0.2148	0.22219
2	metab_127	Mequinol	150-76-5	C7H8O2	Others	0.24064	0.24465	0.26807	0.22971	0.23597	0.23278	0.18165	0.19329	0.19553	0.18441	0.19357	0.1919	0.21962	0.22849	0.2241
2	metab_131	Naphthalene,2,6-dimethoxy-	5486-55-5	C12H12O2	Others	0.07694	0.07534	0.0728	0.08664	0.10698	0.09661	0.06189	0.05867	0.04972	0.03966	0.04742	0.0436	0.06803	0.06591	0.06696
2	metab_1	Paeonol	552-41-0	C9H10O	Phenols	0.32824	0.37689	0.40385	0.29217	0.33931	0.31528	0.20804	0.22674	0.21882	0.10775	0.10717	0.11729	0.28075	0.25754	0.26903

	41			3																
2	metab_150	Phenylacetic Acid	102-16-9	C15H14O2	Acids	0.16567	0.17441	0.11792	0.1406	0.14837	0.14441	0.12088	0.06934	0.10575	0.08787	0.10499	0.08875	0.08754	0.11104	0.09941
2	metab_26	Dihydroactinidiolide	17092-92-1	C11H16O2	Esters	0.50162	0.50919	0.53307	0.52146	0.49984	0.51087	0.42199	0.43528	0.39699	0.3463	0.32298	0.34945	0.34658	0.37313	0.35999
2	metab_38	2-Acetyl-5-methylfuran	1193-79-9	C7H8O2	Heterocyclic compound	0.0571	0.05667	0.06298	0.05515	0.06196	0.05849	0.04236	0.0403	0.04675	0.03646	0.03464	0.03689	0.0405	0.04343	0.04198
2	metab_59	4-Penten-1-ol, propanoate	30563-30-5	C8H14O2	Esters	0.0714	0.07488	0.07803	0.08369	0.07061	0.07728	0.05464	0.05184	0.05883	0.06218	0.06258	0.07754	0.07457	0.06359	0.06902
2	metab_70	Acetophenone	98-86-2	C8H8O	Ketones	0.16447	0.17754	0.18349	0.15688	0.18881	0.17253	0.13786	0.15033	0.14919	0.12467	0.12543	0.13117	0.16181	0.14691	0.15428
2	metab_74	3,4,5-Trimethoxytoluene	6443-69-2	C10H14O3	Benzenes	0.36251	0.4194	0.42213	0.33534	0.43714	0.38524	0.35418	0.37528	0.30953	0.13043	0.12052	0.13822	0.2506	0.24159	0.24605
2	me	Benzene,1,2-dimet	91-	C8	Others	1.04	1.16	1.29	0.99	1.11	1.05	0.73	0.75	0.75	0.60	0.60	0.63	0.99	0.80	0.90

	tab_75	hoxy-	16-7	$H_{10}O_2$		567	292	121	421	62	401	369	253	089	571	017	401	848	646	146
2	metab_84	Butyric Acid	107-92-6	$C_4H_8O_2$	Acids	0.46437	0.46256	0.44875	0.33613	0.33536	0.33575	0.21733	0.22767	0.24501	0.20455	0.20436	0.17107	0.21938	0.16484	0.19182
2	metab_93	(3R,5S)-rel-3,5-Dimethylcyclohexanone	7214-52-0	$C_8H_{14}O$	Ketones	0.05789	0.04939	0.04452	0.05989	0.05302	0.05653	0.03954	0.04044	0.03959	0.02965	0.03333	0.03352	0.04223	0.03922	0.04071
2	metab_94	Cyclopentene	142-29-0	C_5H_8	Hydrocarbons	0.16895	0.16988	0.20092	0.15163	0.16059	0.15603	0.10218	0.10801	0.10837	0.10635	0.10106	0.1177	0.13309	0.14412	0.13866
3	metab_105	2-Acetylpyrrole	1072-83-9	C_6H_7NO	Heterocyclic compound	0.27317	0.24906	0.25977	0.181	0.17978	0.1804	0.20484	0.19689	0.22399	0.25308	0.24759	0.25474	0.20576	0.213	0.20942
3	metab_116	Heptadecane	629-78-7	$C_{17}H_{36}$	Hydrocarbons	0.18211	0.26982	0.1884	0.14114	0.11469	0.12818	0.34312	0.34612	0.28387	0.08481	0.08329	0.07461	0.11549	0.18138	0.14878
3	metab_1	Hexadecane	544-76-3	$C_{16}H_{34}$	Hydrocarbons	0.28973	0.3367	0.28128	0.23695	0.19646	0.2171	0.29685	0.28147	0.29653	0.14107	0.14456	0.13806	0.15551	0.21213	0.18412

	17																			
3	metab_120	4-Methylhexadecane	25117-26-4	$C_{17}H_{36}$	Hydrocarbons	0.2726	0.26089	0.26342	0.02995	0.02919	0.02958	0.21738	0.04181	0.12762	0.02658	0.02399	0.03615	0.03279	0.03469	0.03375
3	metab_136	N-Hexadecanoic acid	1957/10/3	$C_{16}H_{32}O_2$	Acids	1.16696	1.60489	1.73923	0.34451	0.33956	0.34208	0.64375	0.7131	0.66727	0.78114	0.85256	1.08702	0.62204	0.75173	0.68757
3	metab_137	N-Octadecane	593-45-3	$C_{18}H_{38}$	Hydrocarbons	0.06864	0.07842	0.05547	0.0527	0.03957	0.04626	0.18987	0.17265	0.15564	0.04313	0.04507	0.04303	0.05337	0.09235	0.07307
3	metab_154	Phytol	150-86-7	$C_{20}H_{40}O$	Alcohols	0.09594	0.0935	0.10244	0.06337	0.06201	0.0627	0.05994	0.06888	0.06574	0.05943	0.05002	0.06885	0.05256	0.05697	0.05479
3	metab_162	2-Methylpyrazine	109-08-0	$C_5H_6N_2$	Heterocyclic compound	1.67973	1.55965	1.64994	1.30291	1.29303	1.29807	1.38631	1.26793	1.50005	1.77197	1.56939	1.69944	1.40286	1.36138	1.38191
3	metab_164	2-Ethylpyridine	100-71-0	C_7H_9N	Heterocyclic compound	0.84964	0.92106	1.01385	0.23448	0.33404	0.28328	0.31856	0.56944	0.34926	0.24623	0.34876	0.40869	0.44358	0.39514	0.41912
3	me	1H-Pyrazole-4-car	377	C_4	Acids	0.84	0.68	0.51	0.29	0.27	0.28	0.17	0.16	0.16	0.15	0.15	0.14	0.14	0.13	0.14

	tab_17	boxylic acid	18-11-9	H4N2O2		332	209	295	766	786	795	543	9	638	311	596	47	611	928	266
3	metab_32	2,4,5-Trihydroxypyrimidine	496-76-4	C4H4N2O3	Phenols	0.16027	0.15062	0.14708	0.12619	0.12843	0.12729	0.12112	0.13201	0.13084	0.15067	0.14882	0.15601	0.12679	0.13451	0.13069
3	metab_45	2-Methylbutyric Acid	116-53-0	C5H10O2	Acids	0.28323	0.2682	0.27113	0.17533	0.16944	0.17244	0.14171	0.12865	0.14849	0.18939	0.18087	0.16748	0.15135	0.10964	0.13028
3	metab_46	2-Pentadecanone,6,10,14-trimethyl-	502-69-2	C18H36O	Ketones	0.1897	0.19674	0.23183	0.13487	0.12529	0.13018	0.16975	0.17561	0.18142	0.10983	0.10592	0.12181	0.12369	0.13352	0.12866
3	metab_50	3,5-Dihydroxyacetophenone	51863-60-6	C8H8O3	Ketones	0.08399	0.08619	0.09383	0.05475	0.05992	0.05728	0.04802	0.05055	0.04889	0.05247	0.05077	0.05821	0.05688	0.06143	0.05918
3	metab_54	4-(2,6,6-Trimethylcyclohexa-1,3-dienyl)but-3-en-2-one	1203-08-3	C13H18O	Ketones	0.14187	0.13595	0.14418	0.08815	0.09795	0.09295	0.08763	0.09485	0.08148	0.07474	0.07386	0.0813	0.08016	0.08973	0.085
3	metab_7	Benzenemethanol, alpha-[1-(ethylmethylamino)ethyl]-[R	48141-64-	C12H19N	Alcohols	0.41597	0.42016	0.40258	0.34752	0.38741	0.36707	0.43323	0.34756	0.41093	0.43147	0.43108	0.43167	0.30049	0.31001	0.3053

	7	-(R,S)]-	6	O																
4	metab_1	α-Cyclogeraniol acetate	1000158-30-1	$C_{12}H_{20}O_2$	Esters	0.49949	0.52671	0.46941	0.3392	0.359	0.34891	0.33412	0.366	0.34728	0.48727	0.48444	0.46985	0.52065	0.50362	0.51204
4	metab_125	Indole	120-72-9	C_8H_7N	Heterocyclic compound	0.15602	0.15776	0.1828	0.10461	0.12184	0.11305	0.12769	0.12766	0.13601	0.13422	0.15792	0.16516	0.17626	0.19576	0.18612
4	metab_133	Nerol	624-15-7	$C_{10}H_{18}O$	Alcohols	0.50467	0.49324	0.48246	0.29507	0.28457	0.28992	0.33412	0.33243	0.35367	0.54187	0.57837	0.60727	0.56453	0.60441	0.58468
4	metab_134	Neryl Acetate	141-12-8	$C_{12}H_{20}O_2$	Esters	0.1624	0.15834	0.14312	0.10762	0.12921	0.1182	0.10615	0.10986	0.11156	0.1487	0.16506	0.17524	0.16205	0.17294	0.16755
4	metab_145	Phenol	108-95-2	C_6H_6O	Phenols	0.36354	0.37619	0.39656	0.2689	0.27654	0.27265	0.29217	0.29005	0.31511	0.30244	0.34413	0.33459	0.37727	0.39817	0.38783
4	metab_151	Phenylethanol	1960/12/8	$C_8H_{10}O$	Alcohols	0.60533	0.62989	0.73384	0.22386	0.21496	0.2195	0.34815	0.36803	0.39386	0.49972	0.45828	0.50891	0.61609	0.5916	0.60372
4	me	α-Phellandrene	99-	C10	Hydro	0.05	0.05	0.05	0.03	0.02	0.02	0.03	0.03	0.03	0.06	0.06	0.07	0.06	0.07	0.06

	tab_2		83-2	H16	carbons	76	479	362	082	883	984	81	854	997	218	874	276	167	296	737
4	metab_40	2-Butanone	78-93-3	C4H8O	Ketones	0.10474	0.10516	0.10276	0.11793	0.11463	0.11631	0.08757	0.08575	0.08171	0.06042	0.06593	0.06453	0.53599	0.05662	0.29378
4	metab_42	cis-alpha,alpha,5-trimethyl-5-vinyltetrahydrofuran-2-methanol	5989-33-3	C10H18O2	Alcohols	1.3193	1.32242	1.45975	1.17569	1.23033	1.20247	1.00645	1.09864	1.15219	1.20207	1.1273	1.2999	1.34637	1.21464	1.27981
4	metab_53	3-Hydroxypyridine monoacetate	17747-43-2	C7H7NO2	Heterocyclic compound	0.03599	0.03837	0.03682	0.02514	0.02553	0.02533	0.02751	0.02512	0.02884	0.03078	0.03477	0.03902	0.39993	0.03332	0.21469
4	metab_64	Acetamide	60-35-5	C2H5NO	Others	0.26179	0.2559	0.25227	0.26167	0.25591	0.25885	0.23904	0.26599	0.26569	0.27166	0.29409	0.28096	0.29603	0.29644	0.29624
4	metab_78	Benzofuran,2,3-dihydro-	496-16-2	C8H8O	Hydrocarbons	0.34629	0.33281	0.35133	0.25009	0.29366	0.27145	0.27407	0.29446	0.25018	0.32772	0.34135	0.3982	0.31768	0.39106	0.35476
5	metab_102	D-Limonene	5989-27-5	C10H16	Hydrocarbons	0.1336	0.14129	0.12648	0.19239	0.2608	0.22592	0.03979	0.04083	0.04246	0.05979	0.06438	0.06551	0.05615	0.05968	0.05794

5	metab_110	2-ethylfuran	3208-16-0	C6H8O	Heterocyclic compound	1.07786	1.07418	0.81308	1.3017	1.27548	1.28885	1.04905	1.01979	1.04998	0.75882	0.73154	0.61601	0.72016	0.67603	0.69787
5	metab_111	2-Methylfuran	534-22-5	C5H6O	Heterocyclic compound	1.54292	1.52891	1.42435	1.5893	1.6768	1.63219	1.53601	1.49635	1.62142	1.36868	1.42602	1.29322	1.39751	1.31713	1.35689
5	metab_112	Furfural	1998/1/1	C5H4O2	Aldehydes	0.84677	0.78359	0.75391	1.17388	1.01273	1.09489	0.76682	0.70499	0.80665	0.65398	0.5292	0.54505	0.52499	0.51107	0.51796
5	metab_122	Hexanal	66-25-1	C6H12O	Aldehydes	0.22373	0.23572	0.24081	0.27137	0.24563	0.25875	0.19449	0.18831	0.18932	0.12908	0.11524	0.12953	0.11561	0.12775	0.12174
5	metab_14	1,3-Propanediol	504-63-2	C3H8O2	Alcohols	0.13457	0.13932	0.12046	0.16092	0.15526	0.15815	0.12242	0.52485	0.12712	0.09498	0.09349	0.08993	0.21896	0.10871	0.16326
5	metab_142	Pentamethylbenzene	700-12-9	C11H16	Benzenes	0.0399	0.04405	0.04613	0.07127	0.08409	0.07755	0.07514	0.08024	0.07753	0.05154	0.04514	0.04827	0.05497	0.05754	0.05627
5	metab	Pentyl Furan	3777-6	C9H1	Heterocyclic	0.17976	0.20132	0.17852	0.25166	0.23352	0.24277	0.20111	0.19758	0.20622	0.13687	0.13562	0.12644	0.14742	0.15128	0.14937

	_144		9-3	4O	compound															
5	metab_156	Propionaldehyde	123-38-6	C3H6O	Aldehydes	0.64327	0.62989	0.65987	0.79465	0.76127	0.77829	0.59701	0.68237	0.59932	0.47278	0.85856	0.74714	0.41634	0.52301	0.47024
5	metab_157	Propionic Acid	29102	C3H6O2	Acids	0.97726	0.98602	0.92297	1.13409	1.07983	1.10749	0.77392	0.7073	0.78458	0.75259	0.68303	0.61807	0.68746	0.58869	0.63755
5	metab_158	Propiophenone	93-55-0	C9H10O	Ketones	0.35681	0.42526	0.37801	0.44936	0.45614	0.45268	0.31407	0.29997	0.3107	0.16448	0.14871	0.15693	0.16443	0.17492	0.16973
5	metab_21	Pyrrole-2-carboxaldehyde	1003-29-8	C5H5NO	Aldehydes	1.64984	1.62229	1.4608	1.92814	1.68621	1.80955	1.5863	1.52998	1.54883	1.44965	1.18181	1.25414	1.27322	1.23502	1.25392
5	metab_24	5-Methoxy-1-pentene	1191-31-7	C6H12O	Hydrocarbons	0.24541	0.25509	0.21466	0.38334	0.40528	0.39409	0.47341	0.49424	0.45158	0.2604	0.39237	0.33264	0.41812	0.38699	0.40239
5	metab_31	2,3-Pentanedione	600-14-6	C5H8O2	Ketones	0.20269	0.20637	0.21122	0.28452	0.24832	0.26678	0.20028	0.1843	0.20488	0.19329	0.17531	0.19262	0.13987	0.15529	0.14766

5	metab_41	5-Methyl furfural	620-02-0	C6H6O2	Aldehydes	0.52076	0.51308	0.541	0.66743	0.68916	0.67808	0.47672	0.42131	0.49914	0.36825	0.30325	0.30322	0.29889	0.29283	0.29583
5	metab_49	Isophorone	78-59-1	C9H14O	Ketones	0.16493	0.16942	0.19838	0.28	0.30238	0.29097	0.18952	0.20552	0.21029	0.15472	0.16201	0.1558	0.1639	0.16554	0.16473
5	metab_56	1-(4-Butoxyphenyl) ethanone	5736-89-0	C12H16O2	Ketones	0.06093	0.0616	0.0662	0.10545	0.08754	0.09667	0.05321	0.0502	0.05385	0.05476	0.04171	0.0544	0.04472	0.0469	0.04582
5	metab_6	1,2,3,4-Tetramethoxybenzene	21450-56-6	C10H14O4	Benzenes	0.10836	0.12447	0.12437	0.29881	0.30695	0.3028	0.13863	0.15137	0.14832	0.06358	0.07435	0.0669	0.11263	0.11844	0.11557
5	metab_66	Acetic Acid	109-60-4	C5H10O2	Acids	0.70363	0.66614	0.56689	0.89956	0.86098	0.88065	0.71949	0.51975	0.7236	0.74325	0.76425	0.61447	0.73563	0.63585	0.68521
5	metab_68	Methyl acetate	79-20-9	C3H6O2	Aldehydes	3.07376	3.4847	3.63589	4.50682	4.9721	4.73489	3.50099	3.80263	3.64239	2.10311	2.06871	2.21055	3.62163	3.33066	3.47461
5	metab	Benzaldehyde	100-52-	C7H6	Aldehydes	0.79215	0.86073	0.82524	0.8664	0.87979	0.87296	0.7609	0.74093	0.82349	0.67007	0.63015	0.65972	0.66843	0.73018	0.69963

	_71		7	O										000000000006						
5	metab_83	Isovaleraldehyde	590-86-3	C5H10O	Aldehydes	1.94532	2.25972	1.81478	2.10841	2.05431	2.08189	1.88096	2.59384	1.99135	1.95207	2.048	1.95757	1.63895	1.79402	1.7173
5	metab_92	2,2,6-Trimethylcyclohexanone	2408-37-9	C9H16O	Ketones	0.124	0.12847	0.12347	0.14983	0.15827	0.15397	0.11248	0.11102	0.11307	0.09903000000000007	0.09343	0.10299	0.10074	0.11052	0.10568
5	metab_96	3-Methyl-1-cyclopentene	1120-62-3	C6H10	Hydrocarbons	0.02582	0.03345	0.03038	0.04245	0.04946	0.04588	0.06485	0.0536	0.05867	0.03275	0.02577	0.0265	0.02432	0.03308	0.02874
